# Chikungunya virus emergence in the Lao PDR, 2012–2013

**DOI:** 10.1371/journal.pone.0189879

**Published:** 2017-12-28

**Authors:** Somphavanh Somlor, Khamsing Vongpayloth, Laure Diancourt, Philippe Buchy, Veasna Duong, Darouny Phonekeo, Pakapak Ketmayoon, Phengta Vongphrachanh, Paul T. Brey, Valérie Caro, Yves Buisson, Marc Grandadam

**Affiliations:** 1 Institut de la Francophonie pour la Médecine Tropicale, Vientiane, Lao PDR; 2 Institut Pasteur du Laos, Vientiane, Lao PDR; 3 Institut Pasteur, Pôle de Génotypage des Pathogènes, Paris, France; 4 Institut Pasteur du Cambodge, Phnom Penh, Cambodia; 5 National Center for Laboratory and Epidemiology, Vientiane, Lao PDR; Singapore Immunology Network, SINGAPORE

## Abstract

In May 2012, the first authenticated cases of active chikungunya virus infection were detected in Champasak Province, Southern Laos. Analysis of series of human samples and mosquito specimens collected during the outbreak and over the year that followed the emergence enabled the drawing up of a map of the progression of CHIKV and the establishment of a full genetic characterization of the virus.

## Introduction

Chikungunya virus (CHIKV; *Togaviridae*, genus *alphavirus*) was first identified in Tanzania in the 1950s. Less than a decade later, an outbreak was recorded in Thailand, initially considered as a possible consequence of the introduction of CHIKV from Africa [1; 2].

Genetic variability studies of CHIKV strains evidenced three different genotypes, referred to as Western African, Asian, and Eastern/Central/Southern African (ECSA) genotypes. While only a few complete genome sequences of Asian CHIKV isolates were obtained before 2005, phylogenetic analyses supported the contention that Asian isolates had significantly diversified to form a specific Asian genotype present in Asia since at least the eighteenth century [2]. Historical descriptions of clinical syndromes and epidemiologic reports also support the probable presence of CHIKV in Asia during past centuries [2, 3]. Unlike Western African and Asian genotypes that spread poorly out of their regions of origin, the ECSA genotype has dispersed widely since 2006, from the Indian Ocean islands to Asia and Western countries [4, 5, 6]. Several recent studies in Asia over the last ten years evidenced that the ECSA genotype tends to supplant the Asian genotypes [7, 8, 9]. However, Asian genotypes were maintained, at least in Indonesia, and have recently spread out into Southern Pacific territories, the Caribbean islands, and the continental Americas [9, 10, 11].

Follow up on CHIKV emergence events in different countries offered opportunities to study the evolution of the virus in new environmental conditions [12, 13, 14, 15]. The extension of *Aedes* species into new territories during inter-epidemic periods favored the emergence or re-emergence of CHIKV [4, 5, 6, 10]. Such events have been investigated at the phylogenetic level and reveal micro-evolutionary processes with both common and specific signatures within the viral sequences [6, 12, 13]. Among the markers of genetic viral microevolution, adaptive mutation to specific vector species or possibly associated with viral virulence have been reported in structural and/or non-structural genes.

Since that time, numbers of outbreaks have been recorded in urban centers throughout the Indochinese peninsula, while there is very little evidence to support a possible maintenance of the virus in sylvatic cycles [16, 17, 18]. More recently, the emergence of CHIKV was reported in China and Singapore [19].

Detection and isolation of CHIKV from mosquitoes is increasingly reported during epidemics [20, 21 22, 23]. Virological surveillance in vector populations provides valuable information for vector control monitoring and for the assessment of the co-circulation of other *Aedes*-borne viruses such as dengue virus (DENV).

Despite evidence of a frequent circulation of CHIKV in Southeast Asia, only indirect serological data were available in the Lao PDR. The presence of CHIKV or a closely related virus has been mentioned in the past, but relies only on serological investigations [1, 24]. The risk of exposure for populations living in rural or remote mountainous areas has been estimated to be very low [25]. Other alphaviruses, members of the Semliki Forest virus antigenic complex such as Me Tri or Getah, have been reported in neighboring countries, raising the question of the specificity of the antibodies detected in the former studies [26, 27]. A seroprevalence study conducted in Vientiane City detected no positivity, suggesting that the population, at least in this urban setting, was mostly naïve to CHIKV infection [24].

Based on the report of the National Center for Laboratory and Epidemiology (NCLE), a dengue-like syndrome outbreak that occurred from May to September 2012 was linked to CHIKV, as suggested by detection of the viral genome in some suspected cases [24]. A recent published study highlighted the co-circulation of CHIKV and DENV and the occurrence of CHIKV-DENV coinfection in Champasak Province, Southern Laos [28]. On the other hand, some confirmed cases were reported in Phreah Vihear, Northern Cambodia, close to the Lao border, in late 2011, and some scarce molecular information based only on partial envelope gene sequencing suggested a link between Lao and other Southeast Asian CHIKV strains [24, 28, 29]. Furthermore, no geographic extension of CHIKV from the original outbreak area has yet been determined.

We performed multidisciplinary studies from the early beginning of the emergence of CHIKV in Champasak Province in summer 2012 up to December 2013 when the last case was reported. We describe here the general context of CHIKV emergence and spread and provide genetic information to help understand the origin and evolution of the CHIKV virus in the southern provinces of the Lao PDR.

## Materials and methods

### Human samples

Human samples from Champasak Province were collected on different occasions. In early August 2012, grouped cases of dengue-like syndromes were reported by the district health services in two villages of Moonlapamok, Champasak Province, located at the Laos–Cambodia border [24]. Human cases were recorded by the district health authorities using a case definition adapted from the WHO recommendation used for dengue surveillance (i.e. acute fever ≥38.5°C with at least one companion symptom from the list referred to in the WHO 2009 classification of dengue patients) [30]. Patients matching this case definition in Champasak Province were sampled after informed consent by venous puncture within seven days of fever onset and submitted to CHIKV screening. Some samples were first screened for the presence of anti-CHIKV antibodies and viral genome at the NCLE in Vientiane Capital [24]. Some of these samples (n = 16) were then transferred to the Institut Pasteur du Laos for confirmation and viral isolation assays as described elsewhere [31]. An additional series of 51 CHIKV suspected cases were identified and sampled during a joint field mission involving WHO, NCLE, and IPL staff which was set up in Moonlapamock and Khong Districts from July to September 2012.

In 2013, volunteers sampled during a seroprevalence study held in the districts of Moonlapamock and Khong (see below) and presenting a serological profile compatible with an acute phase of a CHIKV infection (n = 34) were screened for the presence of CHIKV genome sequences. Clinicians from provinces located north of Champasak (i.e. Sekong, Savannakhet, Salavan, Attapeu) were informed of the risk of CHIKV circulation and encouraged to collect early blood samples for a combined CHIKV–DENV investigation. A total of 475 cases matching the CHIKV case definition were collected through this investigative network from June to December 2013.

Dengue-like cases collected by the NCLE (n = 47), negative for active dengue infection markers, were screened for CHIKV genome by RT-PCR at IPL as described by Pastorino *et al*. [31].

### Seroprevalence study in Champasak Province

A seroprevalence study was carried out from March 18^th^ to April 11^th^ 2013 in Moonlapamock and Khong Districts, Champasak Province. Data from the National Institute of Statistics were used to make a random selection of villages and determine the size of the sampled population. The whole populations in Moonlapamock and Khong Districts were 33,111 and 84,449 inhabitants respectively. The minimum sample size has been determined for an expected precision of 5% and a statistical accuracy of 80% using the OPEN EPI program. With an estimated prevalence of 50% in a total population of 670,122 inhabitants living in the area studied, the minimum sample size was 384 volunteers. The EPI WHO program was used to calculate the representative number of villages and the required number of volunteers per village. To be significant, it was necessary to include at least 30 villages with a minimum sample of 13 people in each village.

Microsoft Excel software was used to proceed with a random selection of the villages in the two targeted districts. Among the selected villages, four were drawn twice. Thus the targeted number of volunteers was increased to 26 in these villages. At the end of the process, 22 villages were selected in Khong District and four villages in Moonlapamok District. A standardized questionnaire was administered by field workers to collect data on demographics, past symptoms compatible with a CHIKV infection during the previous year, and treatments. A blood sample (5 ml) was collected after informed consent had been obtained. Anti-chikungunya IgM and IgG were detected by means of in-house ELISA methods described elsewhere [6, 32].

### Ethical statements

The studies protocol and surveillance project were approved by the National Ethics Committee for Health Research of the Ministry of Health of the Lao PDR. All public hospitals’ management committees approved the studies and obtained the agreement of the Ministry of Health for participating in the protocol.

All adult volunteers provided written informed consent. A parent or a legal guardian of any child included in the study signed a consent form on their behalf.

### Chikungunya vector(s) investigation

**Fig 1:**
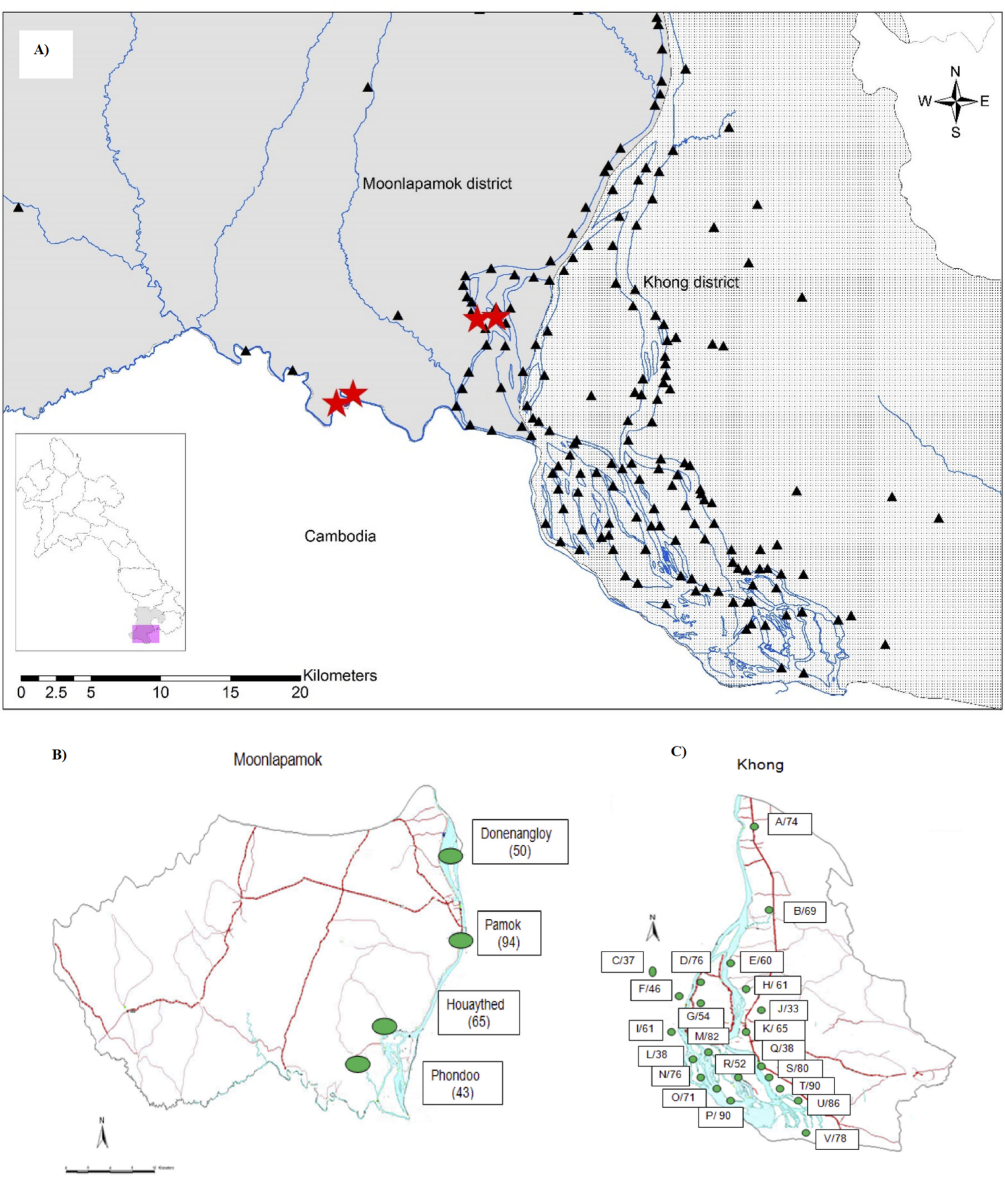
Chikungunya virus study sites in 2012–2013. 1A) Entomologic surveillance sites in September 2012. Black triangles represent villages in Moonlapamok and Khong Districts. Red stars represent villages where mosquito larvae were sampled. 1B) Chikungunya virus IgM seroprevalence in villages in Moonlapamok District. 1C) Chikungunya virus IgM seroprevalence in villages in Khong District. Letters correspond to the villages’ code and numbers to the recorded seroprevalence level.

Entomological surveys were carried out in August 2012 in three villages of Moonlapamok District and one village of Khong District, where suspected and confirmed chikungunya cases were reported (Fig 1A). The houses and neighborhoods of clinically ill patients were selected for larval and adult mosquito sampling. For larval collection, all water-holding containers in and around a house were checked for the presence of immature mosquitoes. Mosquito larvae and pupae were collected using standard 250 ml dippers or sieves and pipettes. The main characteristics of larvae breeding sites (type of container, container function, and maximum capacity) were recorded. The larvae and pupae were counted and reared to adult to improve morphological identification. The House Index (i.e. percentage of houses with *Aedes* breeding sites), Container Index (percentage of containers positive for the presence of *Aedes* larvae), and Breteau Index (positive containers per 100 houses) were calculated.

Adult mosquitoes resting inside a house were collected using sweep nets and aspirators. CDC light traps were set up from 3:30–4:30 pm to 7:30–8:30 am, both inside and outside houses. The mosquito adults (both those that emerged from larvae or nymphs and those collected as adults) were identified morphologically using the keys from Thailand and Vietnam and pooled for virus detection (by species, sex, and method of capture) [33, 34]. Mosquito pools were stored in liquid nitrogen and sent to the Institut Pasteur du Laos in Vientiane Capital.

### Analysis of mosquito samples

Adults or imagoes emerged from larvae were identified and sorted by species and sex. All specimens were dissected individually. Abdomens, wings, and legs of up to ten specimens were grouped in pools for rapid screening. Head-thorax segments were kept frozen at −80°C. Tissue pools were suspended in 400 μl of cold PBS and crushed for 1 min at full speed (25 oscillation/s) in a TissueLyser homogenizer (Qiagen) in the presence of LysingMatrix E beads (MP Biomedicals). Residual tissue fragments were pelleted by spinning the tubes at 10,000g for 5 minutes. One half of the supernatants (200 μl) were used for total nucleic acid extraction using Nucleospin Viral RNA kits (Macherey Nagel) according to manufacturer’s instructions. The rest of the tissue lysates were kept frozen at −80°C for viral isolation assays. Samples were screened for the presence of CHIKV sequences by the real-time RT-PCR method [31].

Head-thorax segments from positive pools were investigated individually by the same procedure to determine the effective number of infected mosquitoes.

### Chikungunya virus isolation

Human samples positive by RT-PCR were inoculated on Vero E6 cells with 100μl of each serum after filtration through a 0.22 μm membrane. Cultures were monitored by daily observation for the presence of cytopathic effect (CPE). Supernatants from cultures displaying a CPE were tested by RT-PCR, generally between Day 3 to Day 5 post-infection. At this stage, CPE generally reached at least 70% of the cell monolayers.

Supernatants of positive pools and head-thorax segments were inoculated to Vero E6 cells. Sub-confluent Vero E6 cells monolayers prepared in 25 cm^2^ flasks were inoculated by 200 μl of supernatant diluted 5 times in DMEM medium after filtration on 0.22μm membranes (Sartorius). The inoculum was removed after 2 hours’ incubation at 37°C and replaced by 5 ml of fresh DMEM completed with 2.5% fetal calf serum. Cultures were monitored using real-time RT-PCR [31].

### Sequence analysis

Viral genomic RNA extraction was carried out from human plasma or from CHIKV primary isolates (passage 1) in Vero E6 cells supernatant using NucleoSpin II RNA kits (Macherey Nagel) according to the manufacturer’s instructions. Sequencing of the E2-6K-E1 region (2,771 nt) or the entire viral genome was performed using primers designed to obtain 700 bp RT-PCR amplicons [6, 12,]. Amplicons generated presented an overlap of 100 bp between contiguous fragments. RT-PCR was performed using SuperScript One-Step RT-PCR with Platinum Taq (Invitrogen) with primers targeting the E2-6K-E1 region or the entire genome [12]. RT-PCR fragments were purified by ultrafiltration. Sequencing reactions were performed using the BigDye Terminator v1.1 cycle sequencing kit (Applied Biosystems). Sequence chromatograms from both strands were obtained on automated sequence analyzer ABI3730XL (Applied Biosystems). For sequence analysis, contig assembly and sequence alignment were performed using the BioNumerics version 6.5 program (Applied-Maths, Saint-Martens-Latem, Belgium).

### Phylogenetic analysis

For phylogenetic analysis of the E2-6K-E1 region or complete genome, maximum-likelihood trees were constructed using MEGA version 6 (www.megasoftware.net), with the Kimura-2 parameter corrections of multiple substitutions. Reliability of nodes was assessed by bootstrap resampling with 5,000 replicates. A sequence of Ivory Coast (West Africa lineage) isolate (HM045818) was used as an outgroup to root the tree. Nucleotide sequences of the E2-6K-E1 region and the complete genome of isolates used in this study were submitted to EMBL-EBI and their accession numbers are shown in Table 1.

**Table 1:** List of Lao CHIKV strains from Champasak Province genetically characterized in this study (with GenBank accession numbers for Lao E2-6K-E1 and complete genome generated sequences).

Isolate ID	District	Village	Date	Acc. Nbr E2-6K-E1	Acc. Nbr Genome
H2013-005-16	Khong	Khone Noy	28/03/2013	LN901359	MF076568
H2013-007-16	Khong	Khone Noy	28/03/2013	LN901362	MF076569
H2013-009-16	Khong	Khone Noy	28/03/2013	LN901356	MF076570
H2013-120	Khong	Loparkchok	26/03/2013	LN901357	
H2013-015	Khong	Huaphiman	29/03/2013	LN901351	
H2013-016	Khong	Huaphiman	26/03/2013	LN901352	
H2013-017	Khong	Huaphiman	30/03/2013	LN901353	
H2013-024	Khong	Huaphiman	30/03/2013	LN901344	
H2012-033	Khong	Donkao	31/08/2012	LN901366	MF076576
H2012-037	Khong	Donkao	31/08/2012	LN901046	
H2013-198	Khong	Loparkchok	26/03/2013	LN901360	
H2013-209	Khong	Loparkchok	26/03/2013	LN901358	
H2013-213	Khong	Loparkchok	26/03/2013	LN901361	
M2012-006P*	Khong	Donkao	31/08/2012	LN901364	MF076577
M2012-5866T*	Khong	Donkao	31/08/2012	LN901365	
M2012-5917T*	Khong	Donkao	31/08/2012	LN901372	
H2013-019-16	Mounlapamok	Luangso	12/03/2013	LN901373	MF076572
H2013-020-16	Mounlapamok	Luangso	12/03/2013	LN901374	
H2013-021-16	Mounlapamok	Luangso	12/03/2013	LN901342	
H2013-002	Mounlapamok	Mai	30/03/2013	LN901343	
H2013-003	Mounlapamok	Mai	31/03/2013	LN901345	
H2013-005	Mounlapamok	Mai	31/03/2013	LN901375	
H2013-006	Mounlapamok	Mai	30/03/2013	LN901376	
H2013-007	Mounlapamok	Mai	30/03/2013	LN901377	
H2013-008	Mounlapamok	Mai	31/03/2013	LN901378	
H2013-009	Mounlapamok	Mai	30/03/2013	LN901379	
H2013-010	Mounlapamok	Mai	01/04/2013	LN901380	
H2013-011	Mounlapamok	Mai	31/03/2013	LN901381	
H2013-012	Mounlapamok	Mai	31/03/2013	LN901382	
H2013-013	Mounlapamok	Mai	31/03/2013	LN901383	
H2013-030	Mounlapamok	Done Nang Loi	31/03/2013	LN901349	
H2012-001	Mounlapamok	Kanleuang	19/07/2012	LN901368	
H2012-003	Mounlapamok	Kanleuang	20/07/2012	LN901367	
H2012-018	Mounlapamok	Nadee	07/08/2012	LN901369	
H2012-019	Mounlapamok	Salaosong	07/08/2012	LN901370	MF076573
H2012-021	Mounlapamok	Huiko	16/08/2012	LN901347	MF076574
H2012-022	Mounlapamok	Nadee	16/08/2012	LN901341	
H2012-028	Mounlapamok	Nadee	16/08/2012	LN901371	MF076575
H2012-029	Mounlapamok	Nadee	16/08/2001	LN901354	
H2013-445	Pakse	Songse	25/12/2013	LN901048	
H2013-011-16	Pathoumphone	Bangliengnai	28/03/2013	LN901363	
H2013-013-16	Pathoumphone	Bangliengnai	28/03/2013	LN901355	MF076571
H2013-025-16	Soukhoumma	Soukhoummatai	18/03/2013	LN901350	

* Mosquito isolates (P = Pool and T = Thorax Head).

## Results

### Detection of Chikungunya virus markers in acute human cases

During the initial investigation of the outbreak from May to July 2012, 52 suspected cases were investigated by the NCLE. A direct diagnosis by RT-PCR could be established for 16 patients (31%) [24]. From these 16 samples referred to the Institut Pasteur du Laos for viral culture assays, 14 CHIKV isolates were obtained.

After the initial alert, a joint field mission involving WHO, NCLE, and IPL staff was set up in the Moonlapamock and Khong Districts. During this period, 51 human suspected cases with clinical profiles compatible with a recent CHIKV infection were recorded in 10 villages located in Moonlapamock and Khong Districts between July 19^th^ and September 10^th^ 2012 (Table 2). All samples were investigated for the presence of anti-CHIKV antibodies. Serological analysis evidenced recent exposure profile (i.e. presence of anti-CHIKV IgM) in 21 of the samples tested negative by RT-PCR. Only one patient was also found positive for anti-CHIKV IgG. Altogether in this initial series, 37 suspected cases (72.5%) displayed a marker of recent chikungunya infection confirming its etiological role in the Champasak Province outbreak.

**Table 2:** Virologic markers of CHIKV infection recorded in suspected cases recorded during the initial field investigation in August 2012.

Villages (n = 51)	District	Date of collection	IFI (IgM/IgG)	RT-PCR
Kanleuang (6)	Moonlapamok	19/07/2012	3	2
Nadee (13)	Moonlapamok	07–16/08/2012	4	4
Salaosong (1)	Moonlapamok	07/08/2012	0	1
Houayko (3)	Moonlapamok	16–17/08/2012	0	1
Donkao (6)	Khong	31/08/2012	3	2
Thakang (7)	Moonlapamok	24–26/08/2012	6†	0
Done hed (4)	Khong	01/09/2012	3	0
Thangsadam (7)	Khong	06/09/2012	2	4
Veunkham (3)	Khong	06–07/09/2012	0	2
Nafang (1)	Khong	10/09/2012	0	0

^†^ 1 case was found positive for IgM and IgG.

In 2013, a major dengue epidemic hit all the provinces of the Lao PDR. Differential diagnosis to discriminate dengue from possible chikungunya infection was applied on samples collected in southern Lao provinces. A total of 485 human samples were tested for the presence of CHIKV genome by real time RT-PCR of which 33 (6.8%) were found positive.

From an independent series of 47 samples of dengue suspected cases recorded in Champasak Province, all found negative for any direct markers of dengue infection by the NCLE, the CHIKV genome was detected in 19 samples (40.4%).

Among the 568 samples collected for the seroprevalence study, 34 (6.0%) showed a serological profile compatible with an acute CHIKV infection, of which two (5.9%) were CHIKV positive by real-time RT-PCR and yielded a viral isolate in culture.

Altogether, a total of 69 CHIKV isolates were obtained from these different series.

### Chikungunya virus seroprevalence in Champasak Province

A total of 568 volunteers were recruited in 26 villages, of which 4 were located in Moonlapamock District and 22 in Khong District of Champasak Province. Of the volunteers, 158 were male (sex ratio M/F: 1/ 2.6), aged 45.5 ± 17.6 (range 3–87 years) and mostly farmers (78.2%). The serological survey revealed that all villages randomly selected had been affected by CHIKV with IgM/IgG seroprevalence levels ranging from 43% to 94% in the four villages selected in Moonlapamok District (Fig 1B) and from 33% to 90% in the 22 villages in Khong District (Fig 1C). No significant differences have been found between the two districts.

### Clinical profile of CHIKV infections

**Fig 2:**
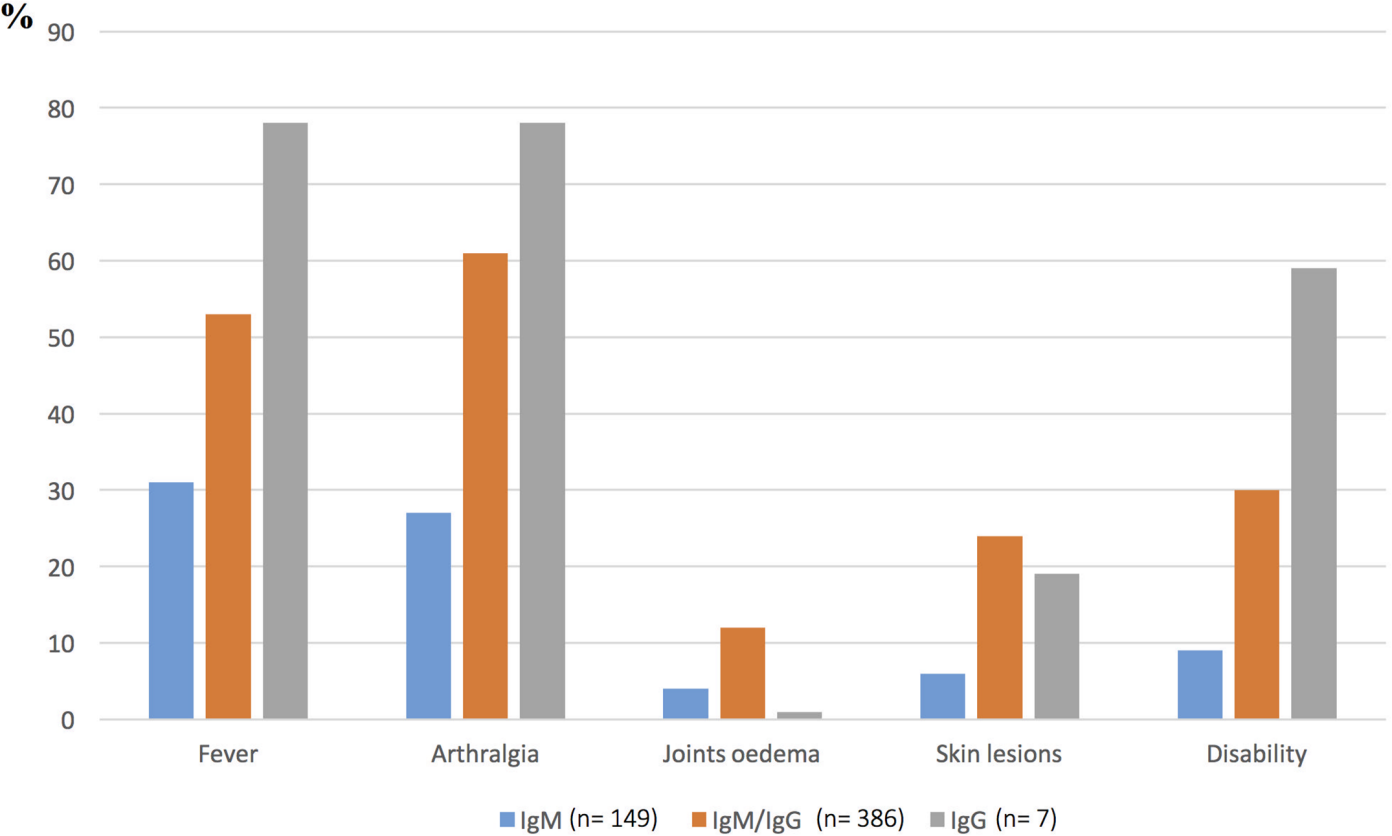
Main symptoms recorded declared by the volunteers during the retrospective seroprevalence study in 2013.

Volunteers were classified into three different groups based on their serological profiles as follows: (i) group 1, presence of anti-CHIKV IgM suggesting recent or possible acute infection stage; (ii) group 2, presence of both IgM and IgG suggestive of a possible convalescent phase; and (iii) group 3, presence of anti-CHIKV IgG only, corresponding to past infection. Among the volunteers positive for at least one immunoglobulin isotype (n = 542), 42.7% declared no clinical history compatible with a CHIKV infection. Most of the participants who experienced symptoms reported an acute fever, often accompanied by severe arthralgia affecting small joints. In summary, the main clinical symptoms recorded in our series were fever, arthralgia, and skin rash. Fig 2 summarizes the main symptoms reported by the participants displaying different anti-CHIKV serologic profiles. Disability, mainly associated with severe recurrent arthralgia, was declared by nearly 60% of the volunteers with isolated IgG.

Of the participants classified in group 1 (IgM+; n = 149), only 35.1% declared clinical signs and symptoms such as fever (33.1%) and arthralgia (29.1%). Joint pain was localized equally in fingers (18.9%) and/or wrists (18.9%) and/or hands (18.9%). Lower limb pain affected feet (21%) and ankles (16.2%). Swelling of joints was reported by 3.4% of participants and joint pain when walking in 7.4% of cases. Other main symptoms reported were myalgia (12.8%), back pain (15.5%), headache (21%), eye pain (7.4%), and vomiting (1.2%). Skin signs were reported by 6.8% of group 1 volunteers of whom 97% declared the occurrence of a rash. In this group of 149 volunteers, 18 (12.1%) reported a prolonged general weakness leading to work disablement for 16 (10.7%) of them.

Clinical signs and symptoms were reported by 65.5% of participants classified in group 2 (i.e. recent infection suggested by positive IgM and IgG; n = 386). Fever and arthralgia were reported by 56.2% and 63.5% respectively of the volunteers. Joint pain was located mainly in the fingers (51%), wrists (51.3%), and hands (48.4%). Lower limb pain affected feet (51.6%) and ankles (45.9%) and swelling of joints was reported by 16.1% of the participants. A significant statistical association was found between this serological profile and headache (34%), asthenia (27.7%), skin rash (26%), back pain (27.7%), myalgias (24.6%), joint pain when walking (24.1%), and eye pain (17.6%). Stoppage of work was declared by 123 volunteers (31.9%). In this group, 19 subjects (4.9%) experienced from 1 to 10 recurrences of arthralgia for a period that never exceeded one month after the acute phase.

Within the last group (past infections IgG+; n = 7), no episode of recurrent arthralgia was reported; the only symptoms statistically associated with this serological profile were ocular pain (80%) and asthenia (60%).

Questionnaire analysis revealed limited population mobility. Only one volunteer (0.7%) reported visiting Cambodia in the neighboring province of Phreah Vihear within the two weeks that preceded the symptom onset, a delay compatible with the CHIKV incubation phase. The possibility of an imported infection, mostly from Cambodia, could be considered for 20 participants (5.2%) as they declared having trading activities in Cambodia but without precise information on dates of stay.

### Entomologic investigation

In the four villages investigated where suspected or confirmed cases were previously reported, high household (81%), container (48%), and Breteau (156) indices were found. Larval breeding sites were found in buckets (1%), drums (2%), tires (7%), discarded household waste (3%), pots (2%), and water jars (85%). Among the different kinds of water containers, water jars used for storing rainwater, with a median volume capacity of 100 liters (range: 2–300 liters) were mostly found positive for *Ae*. *aegypti* larvae, accounting for 84% (3819/4552) of the total larvae collection. As shown in Table 3, the two main vectors of CHIKV were recorded in the different villages. *Ae*. *aegypti* was clearly predominant, representing a mean of 82% of the total population captured (mostly from larvae collection). Among adult mosquitoes, *Ae*. *aegypti* represented 25% of the total population captured by different methods. The CHIKV genome could be detected by RT-PCR in 1 pool of 10 mosquitoes among the 2003 tested. The pools grouped *Aedes aegypti* females captured in Donekhao Village, Moonlapamock District. In order to determine the real number of infected mosquitoes per pool, head-thorax segments preserved were separately analyzed. By this approach, evidence of the CHIKV genome was only found in two mosquitoes within the positive pool. Viral isolation attempted on Vero E6 cells was successful for both the positive specimens.

**Table 3:** Distribution of Chikungunya virus vectors and mosquito species composition from adult and larval collections in villages visited during the initial field investigation in August 2012 (see Fig 1A).

Collection methods/Mosquito species	Khong District	Moonlapamok District			Total	Percentage (%)
	Donkao Village	Kanleung Village	Nadi Village	Thakang Village		
**Adult collection**						
**CDC light traps**						
*Ae*. *aegypti*	Na	Na	4	0	4	0.50
*Ae*. *albopictus*	Na	Na	2	2	4	0.50
*Ae*. *vittatus*	Na	Na	0	1	1	0.12
Other species^b^	Na	Na	189	115	304	37.91
**Total**			**195**	**118**	**313**	39.03
**Sweep nets**						
*Ae*. *aegypti*	46^a^	Na	131	17	194	24.19
*Ae*. *albopictus*	0	Na	0	9	9	1.12
*Ae*. *species*	0	Na	0	9	9	1.12
Other species^b^	96	Na	152	29	277	34.54
**Total**	**142**		**283**	**64**	**489**	60.97
**Total adult collection**	**142**		**478**	**182**	**802**	**100**
**Larvae collection**						
*Ae*. *aegypti*^c^	132	1	875	76	1084	80.84
*Ae*. *albopictus*^c^	37	2	60	68	167	12.45
*Ae*. *vittatus*^c^	0	0	0	6	6	0.45
Other species^b^	82	0	2	0	84	6.26
**Total larvae collection**	**251**	**3**	**937**	**150**	**1341**	**100**
**Total**	**393**	**3**	**1415**	**332**	**2143**	

Na: Not applicable (since trapping was not conducted).

^a^ Two pools of females were positive for CHIKV: One pool of 10 non-engorged females and one pool of one engorged female.

^b^ Other mosquito genera including *Culex* species, *Anopheles* species, *Armigeres* species, and *Mansonia* were also analyzed by RT-PCR for CHIKV but all were negative (132 pools of 286 mosquitoes).

^c^ Species identified after larvae metamorphosis

In order to document a possible vertical transmission of CHIKV in *Aedes* species, the same procedure was applied to reared mosquitoes and adult males but this approach provided no positive identification.

All mosquito pools were also checked for the presence of DENV. Two pools of *Ae aegypti* adult females were found positive for the presence of DENV genome. After individual testing, only one *Aedes* mosquito was found infected in each pool. Typing of DENV by real-time RT-PCR revealed that both isolates belonged to serotype 3. No DENV–CHIKV co-infection could be evidenced in these samples.

### Sequence analysis

**Fig 3:**
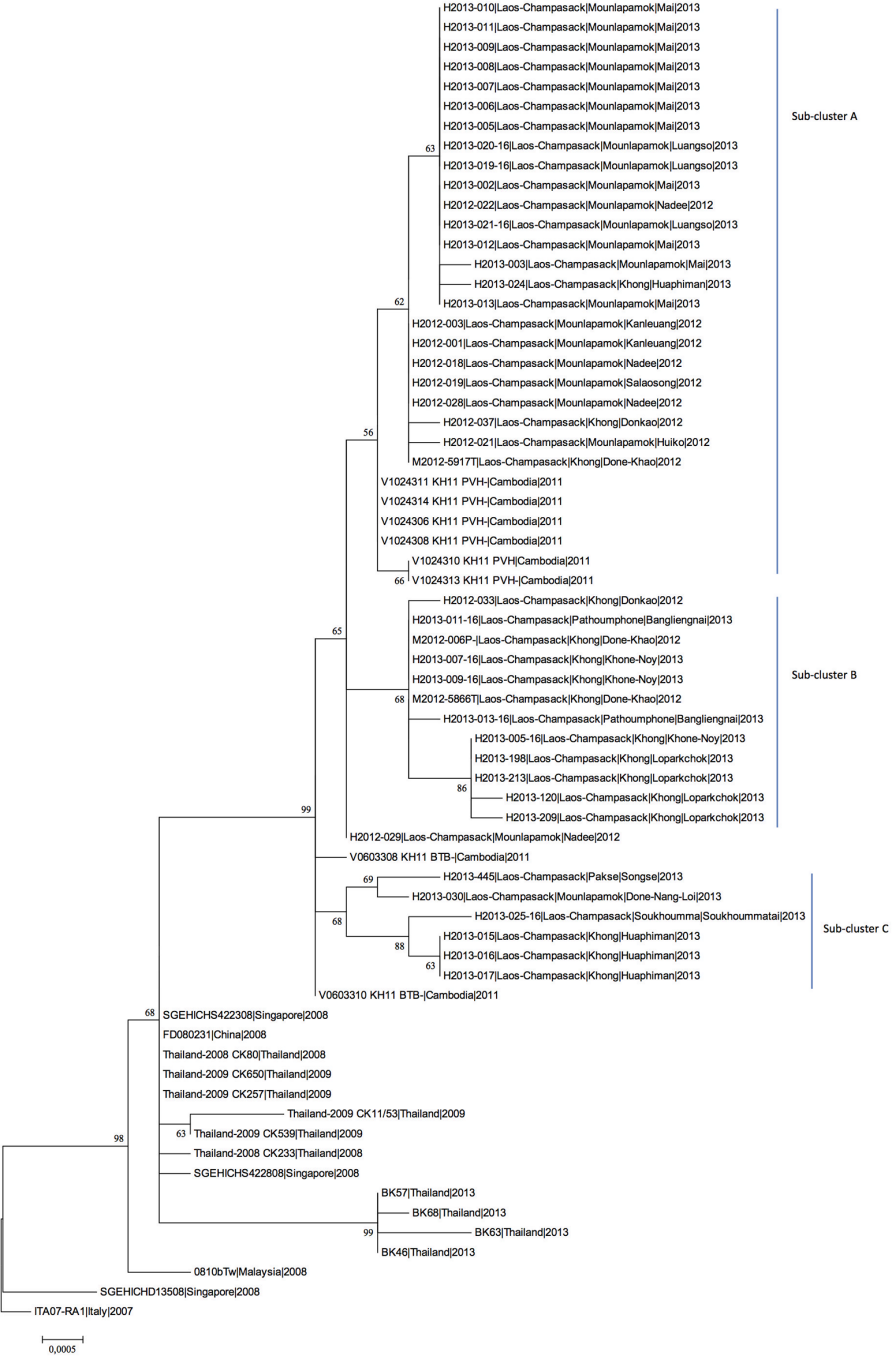
Phylogenetic tree of CHIKV strains based on E2-6K-E1 gene sequences, constructed using the maximum likelihood method.

**Fig 4:**
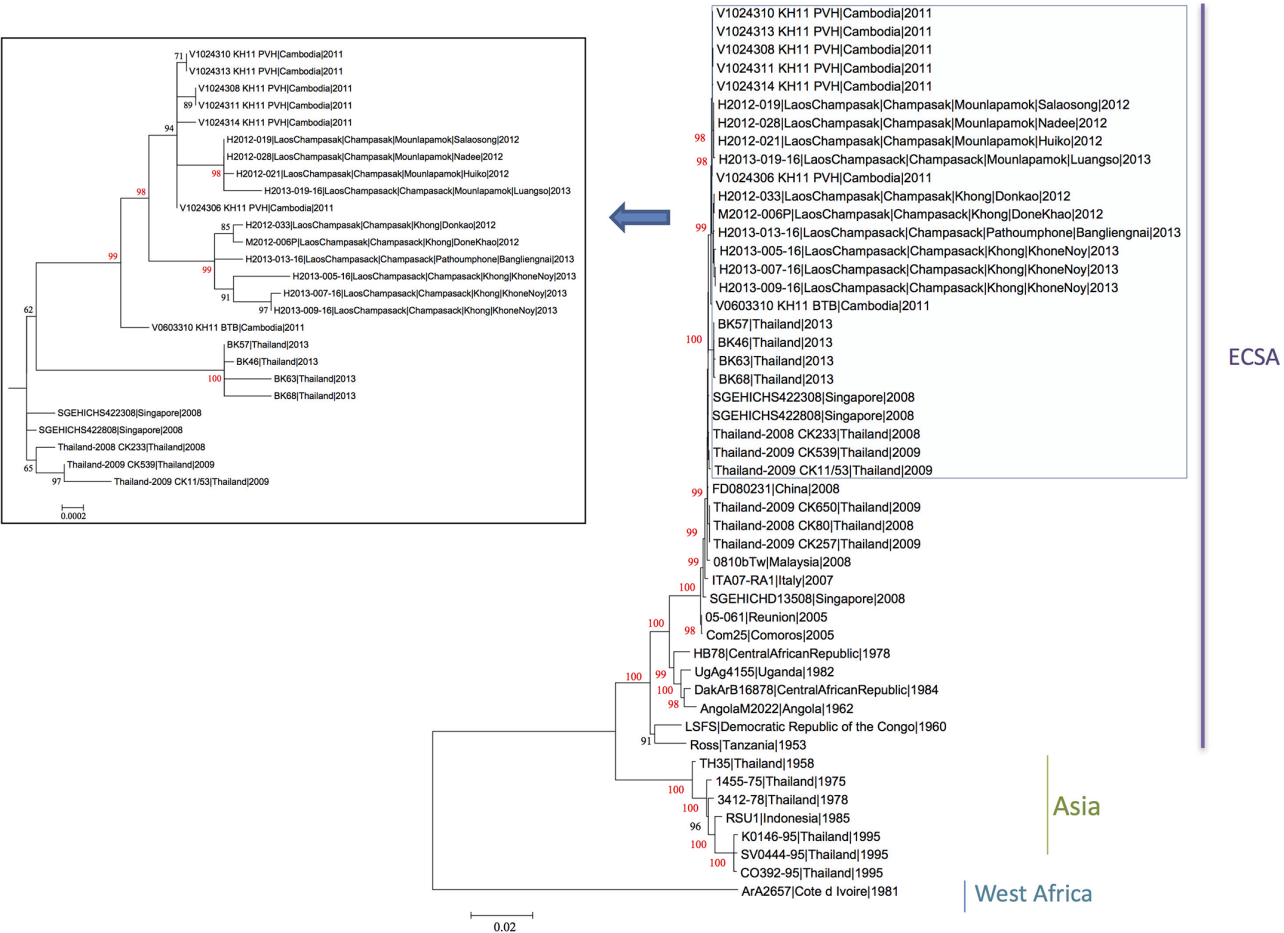
Phylogenetic relationships of CHIKV strains from the Lao PDR based on complete genomes. Phylogenetic tree constructed using the maximum likelihood method.

A total of 69 CHIKV isolates could be obtained among the different series of human and mosquito samples. Of them, 40 human and 3 mosquito isolates (1 from a pool, 2 from individual specimens of the positive pool) were selected for partial or complete genome sequencing. These isolates, obtained from specimens collected between July 2012 and December 2013, were analyzed by sequencing the E2-6K-E1 region (Fig 3). The full genome sequencing has been determined for ten of them (5 from 2012 and 5 from 2013). The phylogenetic tree based on full open reading frames (Fig 4) which identifies the three main phylogroups of CHIKV (West African, East/Central/South African (ECSA) and Asian) showed that all the Lao isolates belonged to the ECSA phylogroup. In this phylogroup, since the major outbreak in the Indian Ocean in 2005, the Indian Ocean lineage (IOL) has spread and diverged in two sub-lineages, Indian Ocean basin and Asian. All the Lao isolates felt in the Asian sub-lineage.

The phylogenetic tree based on 43 Lao E2-6K-E1 sequences, compared with isolates from neighboring countries in Southeast Asia from the same period (Cambodia, China, and Thailand) revealed a cluster between the southern Lao and the Cambodian isolates alone from 2011, well supported by bootstrap value. Interestingly, a mean identity of 99.8% was found between the Lao isolates and the two clusters of strains seen so far in Cambodian provinces (i.e. Battambang, Western Cambodia, and Phreah Vihear, Northern Cambodia near the Lao border) [29]. This analysis also suggests that the Lao isolates could form three different sub-clusters named A, B, and C (Fig 3) without clear correlation with the date and place of collection. This discrimination within the Lao strains, only supported by low bootstrap values when analysis was limited to the E2-6K-E1 segments, could be confirmed by the full genome sequences for the subclusters A and B (bootstraps >98%) for whom full sequence could be established (Fig 4). However, sub-cluster A only comprised the Phreah Vihear (Northern Cambodia) isolates, close to the Lao isolates. The sub-clusters A, B, and C displayed respectively 99.92%, 99.92%, and 99.84% nucleotide identity to each other and harbored specific genetic signatures (Table 4; S1 Table). All these proposed sub-clusters presented the E1-A226V mutation.

**Table 4:** Genetic signatures displayed by the CHIKV Lao isolates over the studied period (August 2012–December 2013).

				nsp1		nsp2				nsp3			nsp4				C		E2	6K
	Key	Country	Date	249	455	130	503	539*	679	330	455	463	76	179	438	604	24	93	252*	60
Sub-cluster A	H2012-019	Laos	2012	**Q**	L	H	I	S	**H**	S	P	H	I	**Y**	V	**V**	T	A	Q	S
	H2012-028	Laos	2012	**Q**	L	H	I	S	**H**	S	P	H	I	**Y**	V	**V**	T	A	Q	S
	H2012-021	Laos	2012	**Q**	L	H	I	S	**H**	S	P	H	I	**Y**	V	**V**	T	A	Q	S
	H2013-019-16	Laos	2013	**Q**	**P**	H	**V**	S	**H**	S	P	**R**	I	**Y**	V	**V**	T	A	Q	S
	V1024310_KH11_PVH	Cambodia	2011	P	L	H	I	S	**H**	S	P	H	I	H	V	I	T	A	Q	S
	V1024313_KH11_PVH	Cambodia	2011	P	L	H	I	S	**H**	S	P	H	I	H	V	I	T	A	Q	S
	V1024306_KH11_PVH	Cambodia	2011	P	L	H	I	S	**H**	S	P	H	I	H	V	I	T	A	Q	S
	V1024308_KH11_PVH	Cambodia	2011	P	L	H	I	S	**H**	S	P	H	I	H	V	I	T	A	Q	S
	V1024311_KH11_PVH	Cambodia	2011	P	L	H	I	S	**H**	S	P	H	I	H	V	I	T	A	Q	S
	V1024314_KH11_PVH	Cambodia	2011	P	L	H	I	S	**H**	S	P	H	I	H	V	I	T	A	Q	S
	V0603310_KH11_BTB	Cambodia	2011	P	L	H	I	S	Y	S	P	H	I	H	V	I	T	A	Q	S
Sub-cluster B	H2012-033	Laos	2012	P	L	**Y**	I	S	Y	**F**	P	H	I	H	V	I	T	**V**	Q	S
	M2012-006	Laos	2012	P	L	**Y**	I	S	Y	**F**	P	H	I	H	V	I	T	**V**	Q	S
	H2013-013-16	Laos	2013	P	L	**Y**	I	S	Y	**F**	**S**	H	I	H	V	I	T	**V**	Q	S
	H2013-007-16	Laos	2013	P	L	**Y**	I	S	Y	**F**	P	H	**V**	H	**D**	I	**A**	**V**	Q	S
	H2013-009-16	Laos	2013	P	L	**Y**	I	S	Y	**F**	P	H	**V**	H	V	I	**A**	**V**	Q	S
	H2013-005-16	Laos	2013	P	L	**Y**	I	S	Y	**F**	P	H	I	H	V	I	**A**	**V**	Q	**N**
	BK46	Thailand	2013	P	L	H	I	S	Y	S	P	H	I	H	V	I	T	A	Q	S
	BK57	Thailand	2013	P	L	H	I	S	Y	S	P	H	I	H	V	I	T	A	Q	S
	BK63	Thailand	2013	P	L	H	I	S	Y	S	P	H	I	H	V	I	T	A	Q	S
	BK68	Thailand	2013	P	L	H	I	S	Y	S	P	H	I	H	V	I	T	A	Q	S
								sl1											sl1	

(*) identifies amino acid residues specific of CHIKV sub-lineage 1.

The consensus sequence obtained from the CHIKV isolated from the mosquito pool (M2012-006P) fell into sub-cluster B and was 100% identical to the subset of human Lao isolates belonging to sub-cluster B.

The E2-6K-E1 sequences of the two isolates obtained from the head-thorax specimens from the positive pool were also established. One isolate (M2012-5866T) was identical to the consensus sequence of the pool (sub-cluster B), but surprisingly, the second one (M2012-5917T) felt into sub-cluster A. The envelope genes sequence of the M2012-5917T isolate shared 99.86% nucleotide identity with the two other mosquito sequences.

Sequencing of the full coding region of the genome was performed for Lao suggested sub-clusters A and B but not in sub-cluster C for which no virus had been isolated from human or mosquito samples. Therefore, a full genome analysis could be achieved for 10 isolates (5 from 2012 and 5 from 2013) in order to better define the position of Lao isolates among the CHIKV lineages from Southeast Asia. The phylogenetic tree based on complete ORF sequences (11,160 nucleotides) revealed the same overall structure as the E2-6K-E1 tree, and at least confirmed the sub-clusters A and B (with strong bootstrap values), with the presence of Lao-Cambodian sub-clusters. Overall homogeneity rates between Lao and Cambodian isolates were 99.84% at the nucleotide and 99.83% at the amino acid levels. These isolates harbored the two mutations nsp2-L539S and E2-K252Q which defined the sub-lineage 1 (sl1) within the IOL [35]. All six Lao isolates from sub-cluster B displayed one specific marker in the structural region C-A93V, and three of the six harbored C-T24A, of which one (isolate H2013-005-16) displayed an additional 6k-S60N substitution, suggesting this sub-cluster was likely in the process of diversification. Isolates within sub-cluster B displayed common signatures in non-structural regions (nsp2-H130Y and nsp3-S330F), with additional markers for three isolates: (i) nsp3-P455S, (ii) nsp4-I76V, and (iii) nsp4-V438D (Table 4; S1 Table).

Interestingly, the four isolates of sub-cluster A shared a common marker (nsp2-Y679H) with all Cambodian isolates. Of these four isolates, none displayed any amino-acid substitution in the structural polyprotein, but only presented some silent mutations in the E2-6K-E1 (6k-nt9860) (4/4), E2-nt9161 (1/4), and E1-nt10931 (1/4) regions (Table 4; S1 Table). In the non-structural regions, four specific markers were recorded in all isolates (i.e. nsp1-P249Q, nsp2-Y679H, nsp4-H179Y, and nsp4-I604V). One isolate harbored additional markers (i.e. nsp1-L455P, nsp2-I503V, nsp3-H463R).

As shown in Fig 3, the Lao isolates grouped in a sub-lineage “Asia” within the ECSA genotype. Close relationships were found with the Cambodian strains. Moreover, the Lao isolates were closely related (99.6% homology) to isolates from Thailand in the same period (2013). Finally, links were found with Chinese (2008: 99.4% homology) and Malaysian (2008: 99.4% homology) strains. Interestingly, one isolate (H2013-013-16) from Pathoumphone Village (in the north of Champasak Province) matching with sub-cluster B displayed some specific markers, such as the amino-acid mutation nsp3-P455S. Moreover, this isolate presented an unambiguous quasi-species at E1-10787 nt position (50%C/50%G analyzed by Sanger sequencing), attesting to the ongoing evolution.

## Discussion

The circulation of CHIKV in the Lao PDR has been suspected for decades based on serological studies but despite the presence of wild and urban vectors, CHIKV had never been isolated in this country [1, 25]. However, the weakness of virology laboratories limited the ability to perform an etiologic diagnosis and may have contributed to confusion between CHIKV and other arboviral infections such as dengue fever. Moreover, the circulation in Southeast Asia of different members of the Semliki Forest virus antigenic complex has been clearly established [26, 27]. Indeed, the presence of anti-CHIKV antibodies reported by different authors may reflect these cryptic (misdiagnosed) infections. However, the low overall rate of seroprevalence in the Lao population leaves the way open for CHIKV to spread in the human population. This threat is confirmed by the fact that most of the patients included in our study displayed markers of recent infection (i.e. positive RT-PCR; presence of IgM). These data evidenced that, despite the official declaration of the end of the epidemic in September 2012, the virus transmission was maintained at a low level in 2013. It is worth noting that the sudden re-emergence of DENV serotype 3 at the beginning of a major outbreak countrywide may have hidden the persistence of CHIKV as a consequence of clinical confusion and/or lack of laboratory diagnosis resources. Previous descriptions of DENV–CHIKV co-infection support these assumptions [28].

Circulation of CHIKV was proven in the Lao PDR in 2012 by the detection of the virus genome in human samples but the real extent of this emergence and especially the phylogenetic position of the virus remained unknown [24]. A large-scale seroprevalence study has been organized to evaluate its true incidence.

Anti-CHIKV antibodies were detected in some groups in the Vientiane City population in the 1960s, but neither the prevalence nor the specificity of these antibodies were specified [25]. A second seroprevalence study performed in Vientiane City in the late 1970s recorded an anti-CHIKV antibody rate of 30% in the general population [36]. Over nearly 40 years, only one more study was conducted in the capital but none of the samples tested (n = 200) were found positive for anti-CHIKV IgG [24]. These results suggest that changes in CHIKV transmission may have occurred in the capital city but could also be linked to the methods used for antibody investigation.

The high proportion of recent infection profiles (isolated IgM: 26.1%) in our study may be related to a low anti-CHIKV immunity level in Lao people. Since the phase of viremia may overlap the rise of IgM antibodies, RT-PCR was tentatively used to detect the presence of CHIKV genome in the plasma of the corresponding cases. Of the 34 samples analyzed, two were found positive by RT-PCR and virus isolation, demonstrating the continued presence of CHIKV several months after the official declaration of the end of the outbreak. Moreover, the combined CHIKV–DENV surveillance in 2013 evidenced the persistence of CHIKV transmission until December 2013.

Among the volunteers included in our study, 42.7% did not report any symptoms suggestive of a CHIKV infection. This rate of asymptomatic infection appears dramatically higher compared to previously published data. A study published in 2008 reported that among the 568 participants, only 3.2% had asymptomatic infections [37]. Among other studies recently reviewed, the maximum asymptomatic rate recorded so far reached 27.7% [38]. However, more recently, an unexpected high rate of CHIKV symptomatic infections (82%) has been recorded in a prospective cohort [39]. In our study, asymptomatic forms were nearly three times higher compared to most of the previous studies but remain lower compared to the last observation in the Philippines. Some bias may explain this unexpected rate. First, a language barrier linked to a specific local dialect and the low educational level of the population may have an impact on the quality of data collection. For example, the difference between symptoms and symptom history may have been misunderstood. Possible cross-reactivity of antibodies with closely related viruses such as Me Tri or Getah with different clinical presentations may have contributed to increasing the rate of asymptomatic cases. And finally, as suggested by Yoon and colleagues, the evaluation of the subclinical CHIKV infection rate could be influenced by the study protocol (prospective *vs* retrospective) [39].

Severe recurrent arthralgia is observed in at least 30% and up to 93% of patients with acute CHIKV infection, whereas in our series only 7.5% of participants reported such episodes [36, 40]. This low rate of recurrent arthralgia may be due to the short delay between the CHIKV emergence and our seroprevalence study. Up to 60% of the participants displaying IgG declared post-acute phase disabilities from 15 days to 6 months following their CHIKV infection, suggesting that, as reported elsewhere, CHIKV may carry serious economic consequences [41, 42]. However, a direct link between CHIKV and these disabilities could not be established with certainty but it could be explained, at least in part, by the harshness of work, as farming is the predominant occupational activity of the population. Thus, a long-term follow up of this exposed population is needed to determine the actual frequency of post-CHIKV arthralgia and evaluate its degree of severity.

In the two districts, the recorded seroprevalence rates ranged from 43% to 94%, revealing the magnitude of the outbreak missed by the preliminary investigations performed in 2012 [24]. These seroprevalence rates give an accurate picture of the herd immunity that may serve as a barrier against future re-emergence (or reintroduction) of CHIKV in the studied area. However, this interpretation should be moderated. Indeed, the higher prevalence observed in females seems to reflect the temporary emigration of males for occupational purposes outside Champasak Province. Thus a substantial proportion of the natives from this region still remained susceptible to CHIKV. Moreover, as no further cases have been diagnosed since December 2013, the level of anti-CHIKV herd immunity in the Lao provinces could be assumed to be very low or non-existent.

Entomologic investigations performed in the two districts in summer 2012 evidenced the presence of the two main recognized vectors of CHIKV, i.e. *Aedes aegypti* and *Aedes albopictus*, with a clear predominance of *Ae*. *aegypti* (82%). However, no correlation could be established between registered entomologic indexes and the anti-CHIKV seroprevalence rates. The vector control campaign implemented a few days before the beginning of the field mission may explain the low vector population densities recorded in the villages. Moreover, only a few villages could be investigated. The disequilibrium observed between the two *Aedes* species vector of CHIKV could also in part be linked to environmental conditions particularly favorable to the development of *Ae*. *aegypti* in the villages where a high infestation rate of jars used for water storage was found. Among many mosquito specimens collected, only *Ae*. *aegypti* yielded positive detection and culture of CHIKV. Comparison of complete viral genome sequences highlighted a strong identity between human and mosquito CHIKV isolates corroborating the vector role of this mosquito species. This does not exclude the possible participation of *Ae*. *albopictus*, as both human and mosquito viral isolates displayed genetic markers of adaptation to *Ae*. *albopictus*, including the E1A226V signature [12, 13]. However, this adaptation process did not occur during the epidemic in Laos as the more closely related 2010–2011 CHIKV isolates from Cambodia already harbored the E1A226V substitution [29]. *Aedes albopictus* has been associated with numbers of recent CHIKV epidemics linked to A226V CHIKV in Southeast Asia whereas *Ae*. *aegypti* has only been involved in wild-type virus transmission [10, 43]. Our findings highlight the potential role of *Ae*. *aegypti* as a vector of ECSA A226V CHIKV in a rural area in the Indochinese peninsula.

As observed in neighboring countries (i.e. Cambodia and Thailand) since the epidemic of 2005 in the Indian Ocean, the CHIKV isolates that circulated in Laos also belonged to the ECSA phylogroup. Interestingly, we could detect and characterize CHIKV isolates from mosquitoes, and the analysis evidenced congruence with the Lao CHIKV human isolates in the same period. More precisely, our analysis shows that all Lao isolates harbor the two specific mutations of the sub-lineage sl1, confirming the filiation of Lao isolates with the Cambodian-Thai cluster and the maintenance of this sub-lineage in this Asian area.

The Lao PDR is surrounded by five countries where chikungunya infection has been reported. There are therefore several possible sources of introduction. Regarding the phylogenetic analysis, the more probable source of CHIKV introduction into the Lao PDR is Cambodia. Sequence comparison allowed the drawing of a clear link between the Lao strains and the Cambodian strains circulating in 2011. Intriguingly, the co-circulation in Laos of different variants previously identified in two remote provinces of Cambodia (i.e. Battambang and Phreah Vihear) raises questions about the introduction pathways of CHIKV. A multiple introduction can be considered, but it cannot be demonstrated due to the low number of strains isolated in Cambodia in 2011 [29]. Another explanation could be that different CHIKV variants co-circulated in Cambodia in the provinces affected by the epidemic, but, as only a few strains were sequenced, only the predominant variant was detected. A recent study of intra-patient genetic diversity confirmed that different genetic variants may co-infect a single individual [44].

Conversely, the possibility of a silent circulation of CHIKV of unknown origin in Laos, before their diffusion to Cambodia, is also a tenable assumption.

Our analysis, including a comparison between isolates from Thailand and Laos in 2013, showed that an introduction from Thailand is highly unlikely.

CHIKV and DENV cocirculation has been evidenced during different outbreaks in Africa and Asia. Moreover, in France, a non-endemic country, the co-emergence of both viruses was reported in 2010 [6, 45]. Occurrences of coinfection in humans and mosquitoes have been reported for decades [23, 28, 46, 47, 48]. CHIKV and DENV co-infection raised concerns that the severity of symptoms may be exacerbated, but such a negative effect has not yet been reported [48].

Recently published data, including our own, provide a general view of the emergence of CHIKV in Laos and shed light on the country’s complex situation [24, 28]. Reinforcement of laboratory diagnostic capacities at the national level are needed, as demonstrated by the high rates of DENV–CHIKV co-infection recorded in a study [28]. The absence of new declared CHIKV cases since December 2013, the limited spread of the virus in the country, and the low level of herd immunity leave the country vulnerable to a large-scale epidemic. As CHIKV still actively circulates in Southeast Asia, it represents, with other viruses such as Zika, a permanent threat for Laos and justifies the maintenance and extension of surveillance systems for arboviral infections.

## Supporting information

S1 Table Complete inventory of genetic signatures displayed by the CHIKV Lao isolates over the studied period (August 2012–December 2013). (XLSX)
